# Cerebral Regional Homogeneity Alternation of Pregnant Women With Antenatal Depression During the Pandemic

**DOI:** 10.3389/fpsyt.2021.627871

**Published:** 2021-04-20

**Authors:** Bochao Cheng, Yajing Meng, Yushan Zhou, Jinrong Li, Jianguang Zeng, Xi Tan, Kaiyou Zhang, Ya Luo, Yan Zhang

**Affiliations:** ^1^Department of Radiology, West China Second University Hospital, Sichuan University, Chengdu, China; ^2^Department of Psychiatry, West China Hospital of Sichuan University, Chengdu, China; ^3^Department of Nuclear Medicine, West China Hospital of Sichuan University, Chengdu, China; ^4^Key Laboratory of Birth Defects and Related Diseases of Women and Children, Ministry of Education, Sichuan University, Chengdu, China; ^5^School of Economics and Business Administration, Chongqing University, Chongqing, China; ^6^Department of Gynecology and Obstetrics, West China Second University Hospital, Sichuan University, Chengdu, China; ^7^Department of Clinical Laboratory, West China Second Hospital, Sichuan University, Chengdu, China; ^8^Department of Gynecology and Obstetrics, Wuxi Maternal and Child Health Hospital, The Affiliated Hospital of Nanjing Medical University, Wuxi, China

**Keywords:** COVID-19, antenatal depression, resting-state fMRI, ReHo, pregnant women, social isolation

## Abstract

**Purpose:** The COVID-19 epidemic has been a threat to the health of people all over the world. Various precautions during COVID-19 in China have kept a large number of people in isolation, and this has inconvenienced and placed enormous stress on pregnant women. Pregnant women are more likely to suffer from antenatal depression (ANDP) with social isolation or low social support. This research aims to investigate the neurobiological mechanisms underlying ANDP, which impedes early detection and intervention in this disorder.

**Methods:** A total of 43 singleton pregnant women who experienced isolation were recruited, including 21 treatment-naïve ANDP patients and 22 healthy pregnant women (HPW). To explore the intrinsic cerebral activity alternations in ANDP using resting-state functional MRI (rsfMRI), we assessed the local regional homogeneity (ReHo) differences in two groups using the voxel-based whole-brain analysis. The correlation between the regional functional abnormalities and clinical variables in ANDP patients was also examined.

**Results:** Compared with HPW, ANDP patients showed decreased ReHo in the left dorsolateral prefrontal cortex, right insular and the cluster coving the right ventral temporal cortex (VTC), amygdala (AMG), and hippocampus (HIP). The Edinburgh Postnatal Depression Scale (EPDS) scores of ANDP patients negatively correlated with the ReHo in the right VTC, AMG, and HIP.

**Conclusion:** Elucidating the neurobiological features of ANDP patients during COVID-19 is crucial for evolving adequate methods for early diagnosis, precaution, and intervention in a future epidemic.

## Introduction

The World Health Organization declared COVID-19 as a pandemic that has swept into at least 185 countries, areas, or territories, and the number of confirmed cases has risen to 118,058,503, including 2,621,046 deaths, by March 12, 2021, (https://www.who.int/emergencies/diseases/novel-coronavirus-2019). To lower the risk of further disease transmission, various precautions have been taken, such as hand washing, wearing face masks, practicing social distancing, isolation of suspected and diagnosed cases, encouraging people to stay at home, lockdown of communities or cities, etc. With a high risk of infectiousness and lethality, COVID-19 has been described as a killer virus by media or social networking tools, which has perpetuated the sense of danger and pushed huge pressure on the public and, particularly, pregnant women, even triggering fear and mental disorders (i.e., panic attacks, anxiety, and depression). Although the COVID-19 situation in China has greatly improved and most people have returned to normal life, these precautionary isolation efforts for COVID-19 in China not only keep most people inconvenienced, but they may have a significant negative impact on pregnant women, especially since their need for social support is great during this period (1). Thus, many pregnant women have declaimed a sense of sadness or stress and have attended hospital.

Pregnant women are more likely to encounter depression and anxiety with social isolation or low social support. Sufficient social support and social contact during pregnancy are considered preventative factors against developing antepartum depression (ANDP) (1, 2). As one of the primarily susceptible mental disorders during the antenatal period (3), antenatal depression (ANDP) is generally characterized by persistent and pervasive feelings of sadness, loss of interest or joylessness, feelings of guilt, and worthlessness, and difficulties concentrating (4). Different studies have verified that the incidence rates of ANDP range from 5 to 25% (5–9), and these rival the rates of gestational diabetes (10) and hypertension (11). Despite the high prevalence of ANPD, treatment rates in the general population remain astonishingly low. It was reported that 12% of general ANDP patients (12) and 5% of high-risk pregnant women receive psychiatric treatment (13). Thus, ANDP has caused great adverse consequences not only for the well-being of mothers but also for their offspring (14–16). In addition, ANDP has been recognized as the most important independent risk factor for developing postpartum depression (PPD) (17).

As is known, pregnancy causes significant changes in the hormonal state, leading to physiological and biochemical changes in the body. However, sometimes the symptoms of ANDP are atypical, such as loss of appetite, fatigue, and sleep disturbances (18), which has lead to confusion with a regular pregnancy response (19, 20). The underlying neural mechanism of ANDP is still unclear, which impedes early detection and treatment.

Neuroimaging tools have the potential to elucidate the neurological pathologies of psychiatric disorders (21). Fundamental studies have revealed the function and functional connectivity (FC) alterations of the brain during resting-state (22, 23) and different task performances in PPD (24–28). In particular, such interest is mainly concerned with some frequently mentioned regions regarding the prefrontal-limbic neural system covering the dorsolateral prefrontal cortex (DLPFC), orbitofrontal cortex (OFC), medial prefrontal cortex (MPFC), cingulate cortex (CC), temporal cortices, and limbic structures (amygdala (AMG); hippocampus (HIP); striatum) (22, 23, 29, 30). However, the current ANDP research is still at an early stage, and few fMRIs have evaluated the cerebral functional alternation. Only one previous study from our team reported functional divergence in ANDP vs. healthy postpartum women during the non-epidemic period using the method of fractional amplitude of low-frequency fluctuations (fALFF) based on resting-state fMRI (31). The results indicated the abnormal fALFF regions of ANDP concerning dysfunction multiply in the nervous system without any external interference (such as the isolation and stress from COVID-19). Besides, the fALFF value only reflects the regional properties of spontaneous brain activity (32, 33) and hardly reflect the temporal homogeneity of neural activity. On the contrary, the local regional homogeneity (ReHo) of resting-state fMRI signals could reflect the temporal synchronized neural activity of the regional fMRI BOLD signals (34). The ReHo is neurobiologically relevant and dependent upon a combination of anatomical, developmental, and neurocognitive factors. Abnormal ReHo is assumed to be relevant to aberrant changes in the temporal aspects of the spontaneous neural activity in the regional brain and can be used to find abnormal activity in the whole brain (35).

Therefore, we utilized ReHo in the present study to identify the differences between the first episode, treatment-naïve ANDP, and healthy pregnant women (HPW) in China during the COVID-19 pandemic. The correlation between clinical variables and functional abnormalities was also checked in ANDP patients. We hypothesized that ANDP patients have ReHo abnormalities in some structures of the prefrontal-limbic circuit concerning the dysfunction of cognitive control, fear, and stress response.

## Methods

### Participants

This study was approved by the local ethics committee of the West China Second University Hospital of Sichuan University and followed the Helsinki Declaration. All the participants provided written informed consent. Participants were pregnant women who stay at home for isolation for more than 1 month without COVID-19 infection and self-reported sadness or stress during the COVID-19 pandemic. A total of 48 right-handed, pregnant Han women of Chinese nationality were consecutively recruited at the maternity clinic during their second or third trimester of pregnancy (prenatal visits ≧ 24 pregnancy weeks, ages from 25 to 32). Of these, 3 ANDP patients and 2 HPW were excluded due to poor image quality. Finally, 21 pregnant women with ANDP and 22 HPW were included in our study.

The diagnosis of first-episode, treatment-naïve ANDP was made according to the criteria of unipolar major depression of the Structured Clinical Interview for DSM-IV Axis I Disorders (SCID) (36) by two experienced psychiatrists (YL and YJ) at the departments of psychiatry, West China Hospital of Sichuan University. The demographic and clinical characteristics and reproductive information of the women were summarized in Table 1. The exclusion criteria included medical diseases such as cardiovascular diseases and diabetes, a history of depression or anxiety or any other Axis I mental disorders such as schizophrenia, bipolar disorder, and substance dependence (other than nicotine), a history of suicide, alcohol or drug abuse, a history of hormonal contraception, and the use of psychotropic medications or using vasoactive medications or cognitive behavior therapy.

**Table 1:** Demographic information.

	**ANDP patients (*n* = 21)**	**HW (*n* = 22)**
Age	28.36 ± 2.18	28.62 ± 2.06
Education (years)	16.75 ± 6.6	16.61 ± 7.0
Married or cohabiting	93%	92.7%
Urban residents	73%	69%
Employed	82%	79%
**Family income (annual)**		
<100 thousand RMB/year	24.1%	24.3%
100–200 thousand RMB/year	38.6%	38.4%
>200 thousand RMB/year	37.3%	37.3%
**Healthy information**		
Pregnancy (weeks)	27.13 ± 4.51	27.34 ± 4.36
History of bad pregnancy and maternity	24.7%	22.9%
BMI	25.6 ± 2.8	23.2 ± 3.5
EPDS^*^	15.82 ± 4.46	4.87 ± 2.16
BAI^*^	40.6 ± 11.05	29.63 ± 6.38
CD-RISC	60.76 ± 17.86	62.76 ± 15.26
SSRS	13.16 ± 2.96	13.90 ± 2.65

*ANDP, antenatal depression; HW, healthy pregnant women; Anx, anxiety; BAI, Beck Anxiety Inventory; Dep, depression; EPDS, Edinburgh Postnatal Depression Scale; CD-RISC, Chinese Connor–Davidson Resilience Scale; SSRS, Social Support Rating Scale; BMI (kg/m2), Body mass index*.

* *p < 0.05*.

### Questionnaire Measure

The Edinburgh Postnatal Depression Scale (EPDS) (37) is the most validated and widely used self-report screening scale for evaluating depression during the perinatal period. It has been well-validated in the PPD and in the ANDP (38). A total score of ≧13 indicates clinically significant depression (38). Beck's Anxiety Inventory (BAI) (39) is a series of self-report questionnaires used to evaluate the severity of depression and anxiety symptoms, respectively.

The Chinese Connor–Davidson Resilience Scale (CD-RISC) is used to assess an individual's ability to respond and adapt to life adversities, traumas, tragedies, threats, or other major life stresses (40, 41). Social support level was measured with the Social Support Rating Scale (SSRS), which has been applied in a wide range of Chinese populations (42–44) especially in prenatal and postpartum mothers (43, 44).

### Image Acquisition

All participants were scanned by a 3-T magnetic resonance imaging system with a 32-channel head coil (MAGNETOM Skyra, Siemens Medical Solutions, Erlangen, Germany). The rsfMRI images were obtained using a gradient-echo echo-planar imaging (EPI) sequence with the following parameters: repetition time (TR) = 2,000 ms; echo time (TE) = 30 ms; flip angle (FA) = 90°, number of slices = 30; slice thickness = 5 mm; field of view (FOV) = 240 × 240 mm^2^; matrix = 64 × 64; and voxel size = 3.75 × 3.75 × 5 mm^3^. Each scan lasted for 400 seconds (i.e., 200 volumes).

### Data Preprocessing

Data preprocessing was performed using the SPM8 package (http://www.fil.ion.ucl.ac.uk/spm) and DPARSF (45), including slice timing, realignment, and normalization to the Montreal Neurological Institute echo-planar imaging template (each voxel was resampled to 3 × 3 × 3 mm^3^). Finally, several nuisance covariates were regressed out, including six head motion parameters, the average signals from white matter, cerebrospinal fluid, and global signals. ReHo shows similarity or synchronization of fMRI signals of nearest neighboring voxels, and Kendall's coefficient of concordance (KCC) is used for the measurement based on the regional homogeneity hypothesis. The individual ReHo map was generated in a voxel-wise fashion, and all ReHo maps were smoothed with a Gaussian filter of 4 mm full-width half maximum (FWHM) kernel to manage the anatomical variability that was not compensated for by spatial normalization (34). Finally, ReHo maps for each subject were transformed to Z scores for subsequent analysis.

### Statistical Analysis

Demographical and clinical variables were assessed using one-way ANOVA or chi-square test by Statistical Package for Social Sciences (SPSS, version 20.0). All values in the text are displayed as mean ± standard deviation (SD) unless otherwise stated. To explore the differences between ANDP patients and HPW, a two-sample *t* test was performed on the individual normalized ReHo image in a voxel-by-voxel manner using SPM8 with the whole brain volume, age, and pregnant weeks as covariates. The significance was set at *p* < 0.05 (false discovery rate (FDR)-correction; voxel level and cluster level) after the FDR-correction for multiple comparisons with a minimum cluster size of 80 voxels.

Spearman rank correlation analyses were conducted to examine the relationship between demographic/clinical variables and ReHo abnormalities. We defined ROI as consisting of the voxels in the regions that showed the significant ReHo differences between the ANDP and HPW were defined. These ROIs were selected using the xjView tool in SPM8. The ReHo within these ROIs of patients were extracted using the MarsBar toolbox. Then, the correlation analysis was performed using SPSS between the ReHo in these ROIs and all the demographic and clinical variables.

## Results

### Clinical Variables

No significant differences between ANDP and HPW were found in age, marital status, habitation, education, employment, annual family incomes, medical/reproductive history, including pregnant weeks, multiple births, history of bad pregnancy and maternity, etc. (Table 1). Compared with the HPW group, the ANDP group exhibited superior scores in EPDS and BAI (*p* < 0.05). No difference regarding CD-RISC and SSRS scores was found (Table 1).

### fMRI Results

#### ANDP vs. HPW

**Figure 1:**
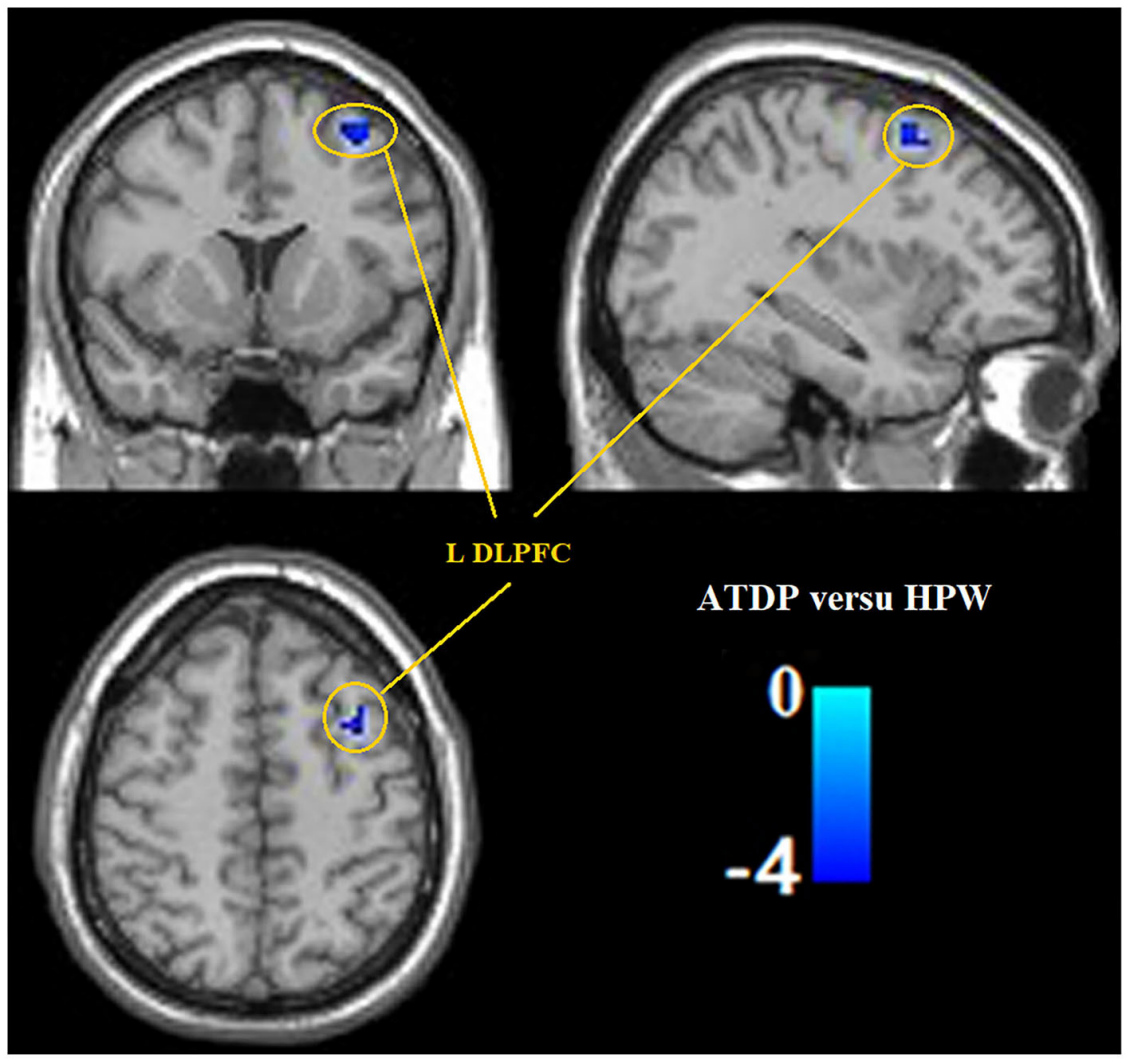
Compared with HPW, ANDP patients showed decreased ReHo in the left DLPFC. Blue to green indicates the regions that exhibit decreased ReHo in ANDP (*p* < 0.05, FDR-corrected). ANDP, Antenatal depression; DLPFC, dorsolateral prefrontal cortex; HPW, healthy pregnant women; ReHo, regional homogeneity; L, left side.

**Figure 2:**
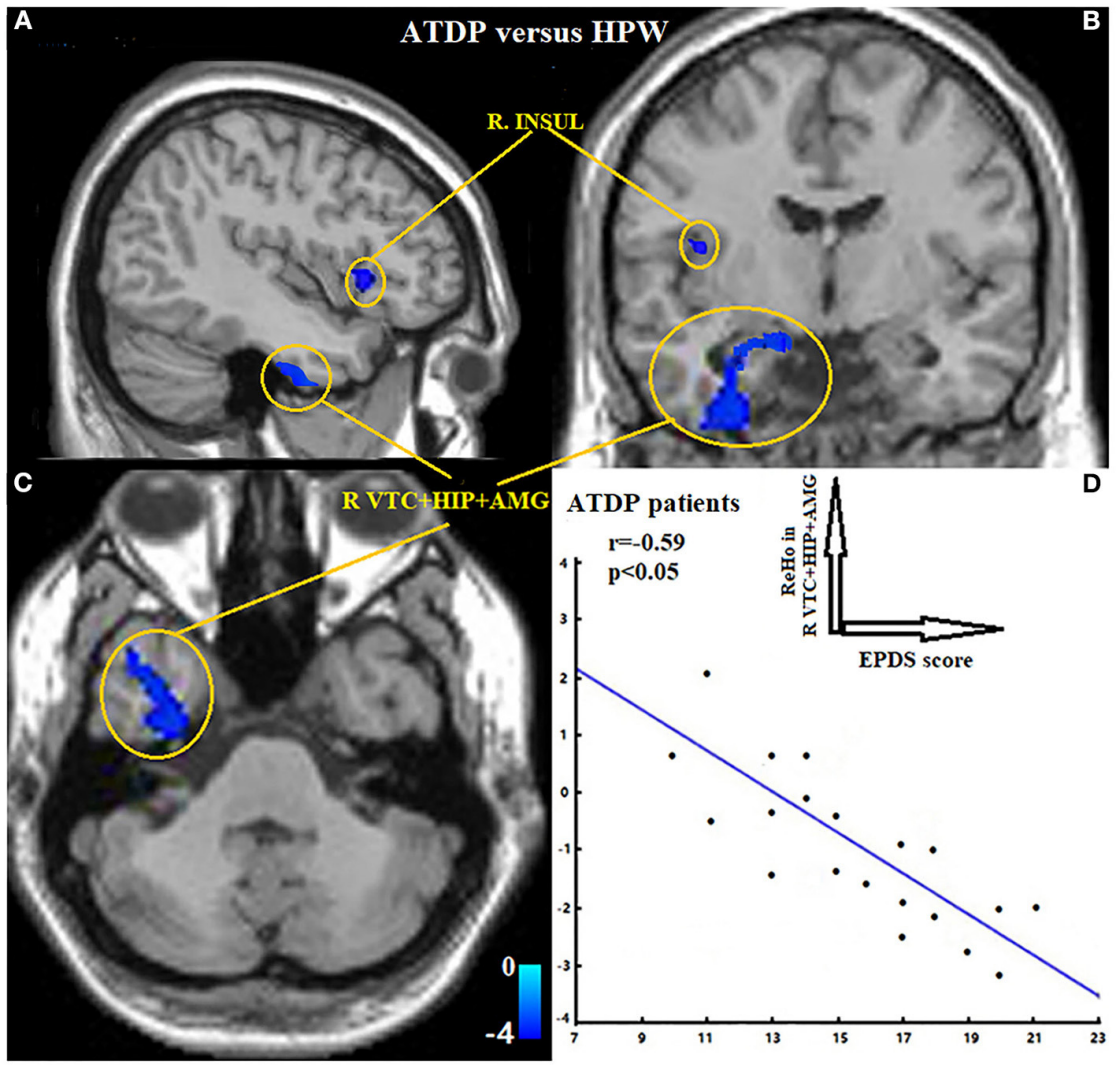
**(A–C)** Exhibited ANDP patients showed decreased ReHo in the right insular, the cluster covering right VTC, HIP, and AMG. Blue to green indicates the regions that exhibit decreased ReHo in ANDP vs. HPW (*p* < 0.05, FDR-corrected). **(D)** Exhibited EPDS scores of ANDP patients were negatively associated with the ReHo of ANDP patients in the right VTC, HIP, and AMG (*r* = −0.59, *p* < 0.05). HPW, healthy pregnant women; EPDS, Edinburgh Postnatal Depression Scale; AMG, amygdala; HIP, hippocampus; INSUL, insular; VTC, ventral temporal cortex. ReHo, regional homogeneity; R, right side.

Compared with the HPW group, the ANDP group had decreased ReHo in the left dorsal prefrontal cortex (DLPFC) (Figure 1 and Table 2) and the cluster covering the right ventral temporal cortices (VTC), HIP, and AMG (Figures 2A–C and Table 2) (*p* < *0.05*, FDR-corrected). No increased ReHo region was found between ANDP vs. HPW.

**Table 2:** Compared with HW, ANDP patients have decreased ReHo regions.

**Location**	**Orientation**	**Alternation**	**Cluster-size (voxels)**	**Peak T-value**	**Primary Peak coordinate (mm)**		
VTC+HIP+AMG	Right	Decrease	265	−3.31	38	−53	−5
Insular	Right	Decrease	94	−3.98	36	−3	−5
DLPFC	Left	Decrease	81	−3.17	−27	38	34

*T-test analysis (whole brain volume, age, and pregnancy time as covariance). AMG, amygdala; ANDP, Antenatal depression; DLPFC, dorsolateral prefrontal cortex; HW, healthy pregnant women; HIP, hippocampus; ReHo, regional homogeneity; VTC, ventral temporal cortex. Cluster-size exceed 80 voxels. P_(FDR−corrected)_ < 0.05*.

#### Correlation

The ReHo values of the region of interests (right VTC, HIP, and AMG) were negatively correlated with the EPDS of the ANDP patients (*r* = −0.59, Figure 2D).

## Discussion

As per previous reports, women are much more vulnerable to stress (46) and showed significantly higher emotional distress than men in isolation during the threat of COVID-19 (47). Yan et al. (48) launched a meta-analysis and revealed the prevalence rates of ANDP during the COVID-19 pandemic are significantly higher than those in the general population (48). Besides, a current longitudinal study across racial and ethnic groups of women during the prenatal and postpartum periods showed that depressive and anxiety symptoms were higher during COVID-19 relative to pre-COVID-19. This increase in depressive symptoms was accompanied by more loneliness (49).

Previous neuroimaging studies have detected functional cerebral abnormalities of non-parturient depressive patients in response to emotional cues in some regions regarding emotion regulation, motivation, and decision making (50, 51). Substantial literature concluded that depression in pregnant women has an impact on their “hedonic” responses to stimuli and may cause maternal executive and cognitive dysfunction (52, 53). Although pregnant women in isolation during the pandemic are not a high-risk group in terms of COVID-19 infection, they suffer greater mental stress, fear and be prone to negative emotions. In our current study, even though no significant difference was detected between the two groups regarding SSRS and CD-RISC scores, the ANDP group exhibited lower CD-RISC and SSRS scores than the HPW group. The results showed ANDP patients have less social support and lower resilience to adapt to life adversities like the COVID-19 pandemic.

The current results confirmed our hypothesis that, under the threat of COVID-19, postpartum women who have intrinsic abnormal cerebral activity in the prefrontal-limbic structures concerning the dysfunction of cognitive control, fear, and stress response develop ANDP. Particularly, these abnormalities include the decrease of ReHo in the left DLPPFC, the right insular, and a cluster covering the right VTC, HIP, and AMG. The higher EPDS scores were also found to be correlated with less ReHo of the right VTC, HIP, and AMG of ANDP patients.

Neuroimaging findings feature the importance of prefrontal cortices (PFC) in parenting behaviors (54). The cognitive system associated with the DLPFC might form such an auxiliary top-down regulation system and regulate the balance between negative and positive emotions (55, 56). The DLPFC could process emotional stimuli (57) and conduct emotion regulation (58). Increased resting-state functional activity (RSFC) has been found in the DLPFC of unhappy people (59). A meta-analysis reported a consistent decreased gray matter volume (GMV) in the ACC, the DLPFC, and dorsomedial prefrontal cortex (DMPFC) of MDD. The DLPFC has also been reported to have a pivotal role in regulating medial PFC/ACC connectivity. After transcranial magnetic stimulation (TMS) of the left DLPFC of depressive patients, an improved working memory performance was observed (60), and this effect is primarily mediated by the medial PFC/ACC, a component of the default mode network (DMN). Combing our results with the abovementioned findings, the evaluated function of DLPFC in ANDP patients may indicate a positive treatment response, whereas the decreased activity of DLPFC may result in impaired cognitive control and emotional regulation.

As a frequently reported limbic structure, AMG is critical for emotional regulation, especially fear and the fear response (61, 62), and has been found to participate in maternal behaviors (63). AMG involves dopamine-associated reward processing and oxytocin release (64) and regulates maternal behavior in other mammals (65). Abler et al. stated that treatment-resist PPD was associated with failing to activate the right amygdala in response to threat-related stimulus (66). In this light, our key finding is that the decreased ReHo of the right AMG of PPD may experience a dilemma in adaptation to the new maternal identity under the threat of COVID-19. This further clarifies amygdala dysregulation associated with ANDP symptomatology, and this is essential to characterize the symptoms of depression that occurs specifically during the perinatal period.

Another limbic area adjunction to AMG, HIP, has been extensively studied in patients with mood disorders. HIP is usually reported to involve learning, memory, and stress responses (67, 68). A reduction of HIP volume has been widely reported in MDD patients (69) and the depressive animal models (70, 71). In addition, extensive rodent and human research have shown that HIP plays an important role in stress responses via the hypothalamus-pituitary-adrenal (HPA) axis. The mnemonic functions and neuroplasticity of HIP are highly sensitive to stress, inducing increased secretion of cortisol (72). Thus, we hypothesize that the long-time exposure to stress and fear (COVID-19 pandemic) may induce the activation of HPA and feedback inhibition of the HIP.

The temporal lobe is critical for memory, emotion, and social information processing, which is a key region closely linked to negative emotion processing. Some studies reported decreased synchronous cerebral activity of depression patients in the right VTC and implicated that the region is strongly linked to the symptoms of PPD patients (22). Our findings reported the hypoactivity activities in the cluster of right VTC, AMG, and HIP of in ANDP patients are accompanied by more serious depressive symptoms, which suggests a decline in the suppression of negative emotions.

As a complex and pivotal brain area taking a vital role in the core limbic system, the insula engages in various cognitive and emotional functions (73). It is also reported to facilitate the interpretation of sensory information that contributes to emotional conditions (74). Insula is also crucial for integrating behavioral and affective processes (75–78). Particularly, insula mediates the relationship between emotional state and decision bias, including maternal love. Its function in processing various sensory input information offers the possibility for inducing subjective feelings (79). The insula of PPD mothers responded less to their own infants' positive expressions (27). In addition, the right insular is involved in self-recognition (80). The hypoactivity of right insular in ANDP patients might indicate difficulty in transition to motherhood and difficulties regarding self-identification.

Given the novelty, the current study also bears several limitations. First is the small sample size of participants. This was owing to the recruitment difficulties in the COVID-19 pandemic, and this may, to some extent, induce statistical errors (false negative and Type II error). Furthermore, our current study only examined the brain regional homogeneity of participants and failed to describe some commonly reported abnormalities such as OFC and cingulate cortex. Small-scale sample sizes, different study designs, and method differences (whole-based vs. ROI) may partly account for this. Third, our study only evaluated antenatal maternity at the second and third trimester and not the first trimester. The fact is that the second and third trimesters during pregnancy are critical periods when neural migration and synaptogenesis of the fetal brain rapidly develop. Moreover, a systematic review reported by Bennett et al. proposed that pregnant women in the second or third trimester have a risk for AD that is twice that of the first trimester (81). Fourth, despite this, behavior studies have demonstrated social isolation may induce a sense of loneliness accompanied by more severe ANDP symptoms. No previous cerebral structural or functional alteration in the isolation situation has been reported. The underlying neural mechanism is still unclear. Therefore, further fMRI studies are needed to address the difference through the comparison of ANDP patients between, during COVID-19, and outside COVID-19. Finally, we only applied resting-state rather than task-based tools, as pregnant women hardly tolerate longtime task-fMRI scanning. Longitudinal studies with multiply fMRI approaches were warranted to recruit a large sample size during the whole pregnancy in order to clarify the underlying neurobiological mechanisms of ANDP.

## Conclusions

In this study, for the first time, we report the changed patterns of spontaneous neural activity in first episode, treatment-naïve ANDP patients during the COVID-19 pandemic. Our results indicated that the aberrant ReHo of ANDP patients is related mainly to prefrontal-limbic circuitry. Defining the neurobiological traits of ANDP patients is necessary for timely diagnosis and treatment, and it may help prevent ANDP in a possible future pandemic.

## Data Availability Statement

The raw data supporting the conclusions of this article will be made available by the authors, without undue reservation.

## Ethics Statement

Written informed consent was obtained from the individual(s) for the publication of any potentially identifiable images or data included in this article.

## Author Contributions

YZha took the main responsibility for study design, initiating, and writing the manuscript. BC and YM contributes to study design, data collection, data analyses, and writing this manuscript. YZho, JL, JZ, XT, KZ, and YL were responsible for the data collection and involved in the enrollment of participants. BC contribute to data analysis and editing the manuscript. All authors have contributed and have approved the final manuscript.

## Conflict of Interest

The authors declare that the research was conducted in the absence of any commercial or financial relationships that could be construed as a potential conflict of interest.
